# Salivary Apelin and Asprosin Levels in Periodontitis and Diabetes Mellitus and Their Relationship with Clinical Periodontal Parameters

**DOI:** 10.3390/diagnostics16071054

**Published:** 2026-04-01

**Authors:** Canan Akdeniz, Arzum Güler Doğru, Revşa Evin Canpolat Erkan

**Affiliations:** 1 Departmentof Periodontology, Faculty of Dentistry, Dicle University, 21280 Diyarbakır, Turkey; 2 Department of Biochemistry, Faculty of Medicine, Dicle University, 21280 Diyarbakır, Turkey

**Keywords:** apelin, asprosin, diabetes mellitus, periodontal health, periodontitis

## Abstract

**Background/Objectives:** Periodontitis and diabetes mellitus (DM) are chronic inflammatory conditions that share common biological mechanisms, including systemic inflammation and insulin resistance. Adipokines are considered key mediators in this interrelationship; however, the roles of many adipokines remain unclear. Apelin and asprosin are relatively novel adipokines that have not yet been sufficiently investigated in dentistry. Therefore, this study aimed to evaluate salivary apelin and asprosin levels in periodontally healthy individuals, patients with periodontitis, and patients with periodontitis + DM and to investigate their associations with clinical periodontal parameters. **Methods:** A total of 90 individuals were included in the study, comprising 30 periodontally healthy subjects, 30 with periodontitis, and 30 with periodontitis and DM. Clinical periodontal indices and body mass index (BMI) were measured for each participant. Unstimulated saliva was collected from each participant, and apelin and asprosin concentrations were analyzed using an enzyme-linked immunosorbent assay (ELISA). The normality of continuous variables was examined with the Shapiro–Wilk test. For non-normally distributed data, non-parametric procedures such as the Mann–Whitney U and Kruskal–Wallis tests were applied. Comparisons of categorical variables between groups were performed using Pearson’s chi-square or the Fisher–Freeman–Halton test. Associations between continuous parameters were assessed through Spearman’s rank correlation analysis. A significance threshold of 5% (*p* < 0.05) was adopted for all statistical evaluations. **Results**: No significant intergroup differences were detected for age, gender, or BMI. The healthy group exhibited significantly lower plaque index (PI), gingival index (GI), and probing depth (PD) scores compared with both periodontitis groups, and these differences reached statistical significance (*p* < 0.001).The median salivary apelin level in the periodontitis + DM group was significantly reduced relative to the healthy group (*p* = 0.009). However, salivary asprosin concentrations did not differ significantly among the groups (*p* = 0.053). Spearman’s correlation analysis revealed positive correlations between asprosin and PD and clinical attachment loss (CAL), whereas apelin showed negative correlations with these parameters. **Conclusions**: Salivary apelin may serve as a potential biomarker for distinguishing healthy individuals from those with diabetic periodontitis. The opposing correlation patterns indicate that apelin and asprosin may be differentially related to periodontal tissue breakdown. However, further longitudinal and mechanistic studies are required to clarify the biological significance of these associations.

## 1. Introduction

Periodontal disease is characterized as a long-standing inflammatory condition with a multifactorial etiology, where gingival inflammation gradually leads to the destruction of tooth-supporting tissues [1]. Its development results from intricate interactions among subgingival microorganisms, the host immune defense, and various environmental determinants [2,3]. DM is a metabolic disease defined by persistent hyperglycemia that arises due to impaired insulin secretion, defective insulin activity, or a combination of both. The pathogenesis of diabetes involves processes such as immune-driven damage to pancreatic β-cells, which ultimately results in inadequate insulin production, as well as peripheral insulin resistance affecting target tissues [4,5].

In the past, periodontitis was regarded as a local inflammatory disease confined to the periodontal tissues. However, over the past four decades, the link connecting periodontal pathology with systemic conditions such as obesity and diabetes has been well established [6,7,8,9]. Some authors have even suggested that, due to this bidirectional association, periodontal disease should no longer be considered a problem limited to a localized anatomical region but rather classified among chronic systemic diseases [10]. A bidirectional relationship exists between periodontal disease and diabetes: poor glycemic control worsens periodontal health, while periodontal inflammation can, in turn, impair glycemic regulation [11]. Likewise, numerous previous studies have indicated a positive relationship between obesity and periodontitis, with obesity increasing both the risk and severity of periodontal disease [12,13,14]. Furthermore, evidence suggests that inflammation originating from periodontitis may worsen metabolic control and contribute to obesity-related complications [15,16]. The key mediators of the relationship among obesity, diabetes, and periodontitis are adipokines—bioactive molecules secreted by adipose tissue that regulate insulin sensitivity and energy metabolism as well as modulate inflammatory and healing processes [17,18]. Adipose tissue, beyond its function as a lipid storage depot, acts as an active endocrine organ capable of synthesizing and releasing numerous metabolically relevant molecules, including various inflammatory mediators. Among these, adipokines play essential roles in regulating insulin sensitivity and energy expenditure, as well as in immune–inflammatory responses, wound healing, and various physiological and pathological processes [19].

In 1993, O’Dowd et al. identified a novel G protein-coupled receptor in humans and named it APJ [20]. Subsequently, in 1998, Tatemoto et al. isolated apelin, the endogenous ligand of APJ, from bovine stomach tissue [21]. Apelin is a regulatory peptide expressed in numerous organs, including the heart, lungs, brain, kidneys, liver, blood vessels, and gastrointestinal system [22]. It was first demonstrated in 2005 that apelin is produced and secreted by adipocytes, after which it began to be recognized as an adipokine [23]. Apelin not only modulates insulin secretion but also plays a role in glucose and lipid metabolism. In addition, it promotes pancreatic β-cell proliferation and supports β-cell survival by reducing apoptosis [24,25]. Through its ability to enhance insulin production and sensitivity, as well as to regulate diabetes-related complications, apelin has emerged as a potential therapeutic target for diabetes management [21,22].

Insulin resistance is commonly associated with obesity, and the inability of pancreatic islets to meet the increased insulin demand predisposes individuals to the development of type 2 DM. The insulin-sensitizing role of apelin has been well documented; however, it remains unclear whether this effect occurs through direct cellular mechanisms, indirect pathways, or a combination of both [26]. Studies demonstrating that apelin reduces lipid accumulation in adipocytes further support this finding [27]. Apelin promotes the formation of brown adipose tissue (BAT) while also inducing the browning of white adipose tissue (WAT). Experimental studies have shown that apelin administration enhances BAT differentiation and increases the expression of brown adipocyte-specific markers in WAT [28]. These effects suggest that apelin may directly contribute to the alleviation of diabetic conditions by regulating energy metabolism.

Asprosin, an adipokine like apelin, was first discovered in 2016 by Romere et al. during genetic studies conducted on patients with neonatal progeroid syndrome [29]. Asprosin is mainly secreted by adipocytes in white adipose tissue; nevertheless, trace expression has also been identified in other organs, including salivary glands, pancreatic β-cell, and cartilage [30]. This hormone originates from the 65th and 66th exons of the fibrillin-1 (FBN1) gene, which is located on chromosome 15q21.1. The primary translation product of FBN1, known as pro-fibrillin-1, consists of 2871 amino acids. FBN1 is proteolytically cleaved by the furin enzyme to generate two products: mature fibrillin 1, which is 2704 amino acids in length, and asprosin, which comprises 140 amino acids [31]. Asprosin is defined as an orexigenic (appetite-stimulating) and glucogenic hormone. It plays a key role in glucose homeostasis by increasing hepatic glucose production during fasting. Elevated circulating asprosin levels have been associated with impaired glucose metabolism and insulin resistance, linking it to obesity and diabetes. Moreover, its orexigenic effects may further contribute to metabolic dysregulation [30,32]. Its appetite-stimulating effect is mediated by central receptors in the hypothalamus, the key region for appetite regulation, which modulates feeding behavior through neurons expressing proopiomelanocortin (POMC) that suppresses appetite and agouti-related peptide (AgRP) that promotes it [33].

Asprosin levels are strongly associated with glucose concentrations, as low glucose levels during fasting stimulate asprosin production, whereas high glucose levels during feeding suppress its secretion [29]. Following the neutralization of asprosin by specific antibodies, improvements in insulin resistance and reductions in blood glucose and insulin levels, as well as decreases in body weight, have been observed [34]. These findings suggest that reducing endogenous asprosin production may have therapeutic potential in the treatment of type 2 DM. Researchers have proposed that circulating asprosin levels could serve as an early diagnostic biomarker for diabetes and as a potential therapeutic target for both prediabetes and type 2 DM [35]. Moreover, studies conducted on systemically diseased mice and humans have reported positive correlations between serum asprosin concentrations and periodontal parameters [36,37].

The association between systemic diseases and periodontitis may be attributed to the low-grade systemic inflammation induced by adipokines, as well as to the systemic effects of inflammatory mediators released from periodontal tissues during periodontitis [38]. Therefore, the aims of the present study were as follows: (1) to evaluate salivary apelin and asprosin levels in patients with periodontitis and periodontitis + DM and compare them with those of healthy individuals; (2) to assess the relationships between asprosin and apelin levels and clinical periodontal indices.

We hypothesized that salivary apelin and asprosin levels would be altered in patients with periodontitis and periodontitis + DM and would be associated with clinical periodontal parameters.

## 2. Materials and Methods

Ethical approval for this study was granted by the Local Ethics Committee of the Faculty of Dentistry, Dicle University (Protocol No: 2024-18; 26 June 2024). Written consent was obtained from all individuals prior to their participation, and the study was carried out in full compliance with international ethical standards, including those stated in the Declaration of Helsinki (1975, revised 2013). This study did not involve any clinical intervention and was not registered as a clinical trial. 

### 2.1. Study Groups

**Figure 1:**
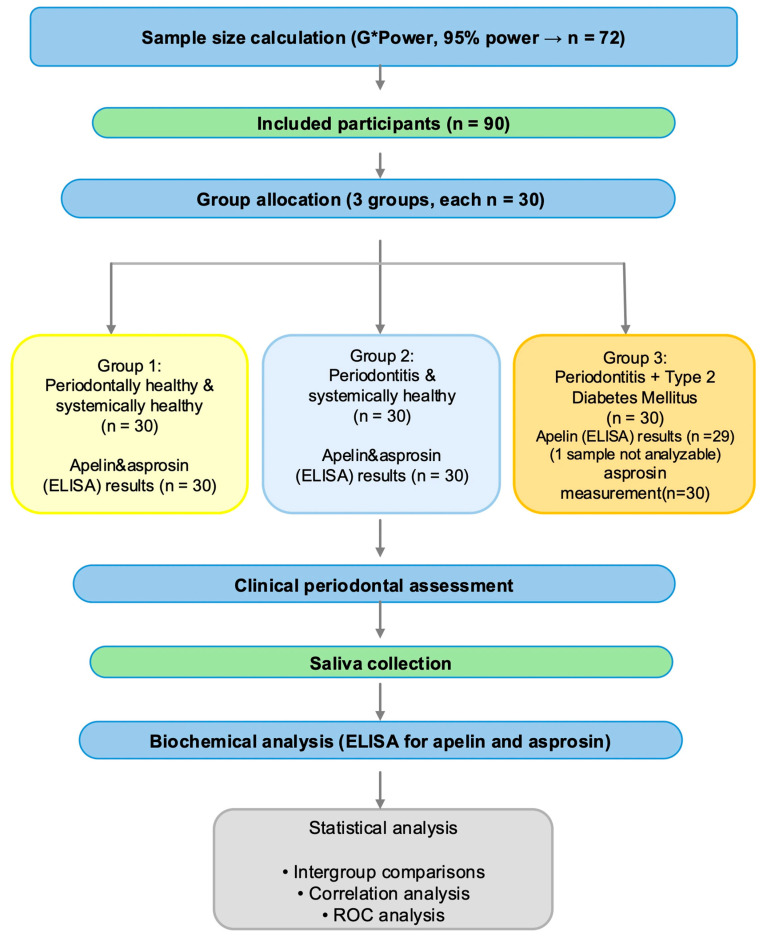
Study design and workflow of clinical, biochemical, and statistical analyses.

A total of 90 voluntary participants who attended the Department of Periodontology, Faculty of Dentistry, Dicle University, were included in the study. Based on a priori power analysis (95% power), a minimum sample size of 72 participants was calculated. However, 90 participants were enrolled to compensate for potential dropouts or unforeseen circumstances. The overall study design and workflow of clinical, biochemical, and statistical analyses are illustrated in Figure 1.

The periodontal status of each subject was assessed based on the most recent classification framework proposed during the 2017 International Consensus Meeting on Periodontal and Peri-Implant Conditions [39].

Group 1 (Periodontally healthy): This group consisted of 30 individuals with less than 10% bleeding on probing, probing depths not exceeding 3 mm, no detectable clinical attachment loss, and no radiographic signs of alveolar bone loss. In addition, none of these participants reported systemic diseases.

Group 2 (Periodontitis): Thirty systemically healthy individuals diagnosed with stage II, III, or IV periodontitis were assigned to this group. The diagnosis was based on the presence of clinical attachment loss at interproximal surfaces of at least two non-adjacent teeth or attachment loss ≥3 mm together with probing depth ≥3 mm on buccal or lingual surfaces of at least two teeth, using six-site-per-tooth measurements.

Group 3 (DM + Periodontitis): This group included 30 patients with type 2 diabetes and stage II–IV periodontitis. Diagnostic criteria were attachment loss at interproximal areas of at least two non-adjacent teeth or probing depth ≥3 mm combined with attachment loss ≥3 mm on the buccal or lingual aspects of at least two teeth, determined from six sites per tooth. To minimize the influence of advanced diabetic complications, only patients with HbA1c levels ranging from 7.0% to 8.5% were included in the diabetic group.

Additional verification of diabetic status was performed through Turkey’s national electronic health record system (e-Nabız) [40]. With participants’ consent, confirmed diagnoses and antidiabetic medication records were reviewed. Only individuals with documented type 2 DM and whose most recent HbA1c values were verified through e-Nabız within the specified range were included in Group 3. BMI was calculated for all participants. To reduce the potential confounding influence of obesity, only individuals classified as having a normal BMI (18.5–24.9 kg/m^2^) according to the World Health Organization criteria were eligible for inclusion in the study [41].

Participants using immunosuppressive medication, pregnant or breastfeeding women, individuals who had received periodontal therapy within the past six months, or smokers were excluded from the study. Additional exclusion criteria comprised the presence of systemic diseases other than diabetes, disorders affecting bone metabolism, antibiotic use within the preceding three months, a history of head and neck radiotherapy within the last six months, and having fewer than 15 remaining teeth.

### 2.2. Clinical Examination

For standardization of periodontal clinical assessments, periodontal parameters were evaluated by one experienced examiner (C.A.) employing a Williams-type probe (Hu-Friedy, Chicago, IL, USA). For each tooth (except third molars), plaque index (PI), gingival index (GI), probing depth (PD), and clinical attachment loss (CAL) values were evaluated at six reference sites: distobuccal, buccal, mesiobuccal, distolingual/distopalatal, lingual/palatal, and mesiolingual/mesiopalatal. To confirm consistency, PD and CAL were reassessed at every site within 24 h by the same calibrated examiner. The consistency of measurements taken by the same examiner was evaluated using intraclass correlation coefficients (ICC), which showed excellent repeatability (ICC = 0.956 for PD and 0.950 for CAL).

### 2.3. Collection of Saliva Samples

Samples of saliva were taken from participants between 09:00 and 11:00 a.m. under standardized morning conditions. Participants were instructed to refrain from eating, drinking (except water), toothbrushing, chewing gum, or using mouthrinses for at least two hours before the appointment. Upon arrival, participants rinsed their mouths thoroughly with water. They were seated comfortably and asked to accumulate unstimulated saliva in the mouth for 10 min, then expectorate into sterile cups. Each saliva sample was centrifuged (10,000 rpm, 5 min), and the isolated supernatant was kept at −80 °C until further evaluation.

### 2.4. Biochemical Analysis

The determination of apelin and asprosin levels in saliva samples was performed using the ELISA method at the Medical Biochemistry Laboratory of the Faculty of Medicine, Dicle University. Apelin concentrations were measured with a commercial ELISA kit (Elabscience^®^ Biotechnology Co., Ltd., Wuhan, China, E-EL-H0456; sensitivity: 0.0375 ng/mL; detection range: 0.0625–4.0 ng/mL), and asprosin levels were determined using another ELISA kit (Elabscience^®^ Biotechnology Co., Ltd., Wuhan, China, E-EL-H0515; sensitivity: 0.19 ng/mL; detection range: 0.31–20 ng/mL). According to the manufacturer, the intra-assay and inter-assay coefficients of variation (CV) for the apelin ELISA kit ranged from 3.70 to 5.90% and 4.20 to 5.38%, respectively, indicating high assay precision. For the asprosin ELISA kit, the intra-assay and inter-assay CV values ranged from 3.98 to 6.67% and 4.15 to 7.14%, respectively.

ELISA analyses were performed following the manufacturer’s protocols.

For the determination of apelin levels, 50 μL of each saliva sample and 50 μL of the biotinylated detection antibody were simultaneously dispensed into the wells and incubated for 45 min at 37 °C. For asprosin, 100 μL of the sample was first incubated for 90 min at 37 °C, after which the wells were emptied and refilled with 100 μL of the biotinylated antibody solution, followed by an additional 60 min incubation at 37 °C. In both assays, subsequent steps included incubation with HRP-conjugate solution, washing, addition of TMB substrate, termination of the reaction with stop solution, and measurement of optical density at 450 nm using a microplate reader ( ELx800; BioTek Instruments, Inc., Winooski, VT, USA).

### 2.5. Statistical Analyses

Sample size estimation was performed using G*Power software (version 3.1.9.7; Heinrich Heine University, Düsseldorf, Germany). An a priori power analysis was conducted for a one-way ANOVA (fixed effects, omnibus test) with a significance level of 0.05 and a statistical power of 95%. Assuming an effect size (f) of 0.475 based on pilot observations and relevant literature, the required total sample size was calculated as 72 participants (24 per group), corresponding to an actual power of 0.9517. When a more conservative effect size (f = 0.449) was considered, the estimated total sample size increased to 81 participants, yielding an actual power of 0.9531.

The distribution of continuous variables was assessed using the Shapiro–Wilk test. Normally distributed data are presented as mean ± standard deviation, whereas non-normally distributed variables are expressed as median (min–max). Categorical variables are summarized as frequency and percentage, *n* (%). For comparisons between two groups, the Mann–Whitney U test was used when normality assumptions were not met. When more than two groups were compared and a normal distribution was not observed, the Kruskal–Wallis test was applied. If overall significance was detected with the Kruskal–Wallis test, post hoc subgroup comparisons were performed using the Dunn–Bonferroni test. Categorical variables were analyzed using Pearson’s chi-square and Fisher–Freeman–Halton tests, as appropriate. Correlation analyses were conducted using Spearman’s rank correlation coefficients. Receiver operating characteristic (ROC) curve analysis was performed to evaluate the diagnostic performance of salivary apelin and asprosin levels. Statistical analyses were carried out using IBM SPSS Statistics software (version 25.0; IBM Corp., Armonk, NY, USA). A *p*-value < 0.05 was considered statistically significant.

## 3. Results

### 3.1. Demographic Features and Clinical Periodontal Parameters

Ninety volunteers, aged 36–49 years, participated in the present study. Demographic data, clinical periodontal parameters, and the mean salivary apelin and asprosin values are presented in Table 1. No significant differences were found among the study groups with respect to age (*p* = 0.158) or gender distribution (*p* = 1.000) (Table 2). In addition, the comparison of BMI values across the groups revealed no statistically significant variation (*p* = 0.645).

PI, GI, and PD scores in the healthy group were considerably lower compared with the other groups, demonstrating statistically significant differences (*p* < 0.001) (Table 2). The CAL value in the periodontitis + DM group was higher than that in the periodontitis group, showing a statistically significant difference (*p* = 0.019) (Table 2).

### 3.2. Comparison of Salivary Asprosin and Apelin Levels Among Groups

**Figure 2:**
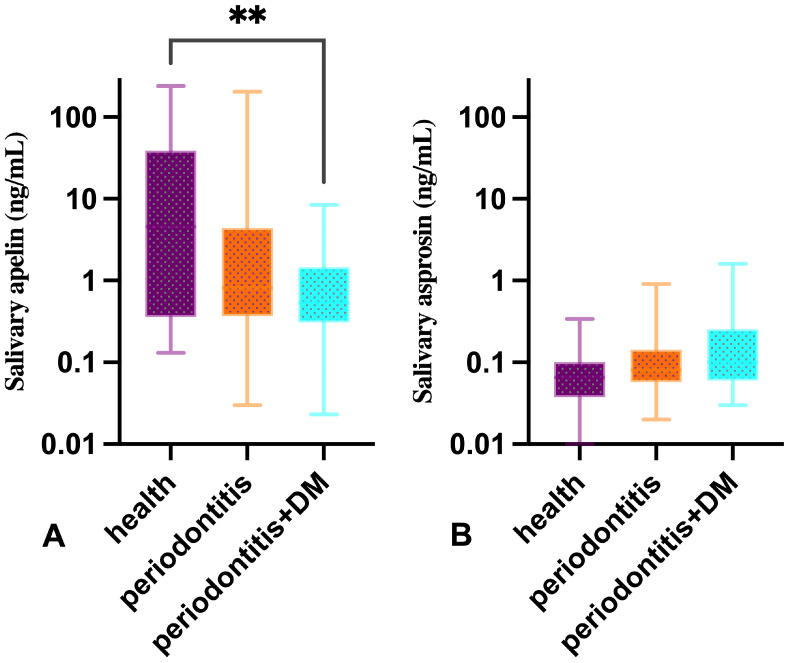
Box-and-whisker plots showing salivary apelin (**A**) and asprosin (**B**) levels in healthy individuals, periodontitis patients, and periodontitis patients with diabetes mellitus. Data are presented on a logarithmic scale (ng/mL). Boxes represent median and interquartile range (IQR), and whiskers indicate minimum–maximum values. A statistically significant difference in salivary apelin levels was observed between healthy individuals and periodontitis patients with diabetes mellitus (*p* = 0.009). ** indicates statistical significance at the *p* < 0.01 level.

In one participant in the periodontitis + DM group, salivary apelin could not be detected because the level was below the detection limit of the ELISA assay. The median values of salivary apelin were measured as 4.56 ng/mL (Q1: 0.36–Q3: 38.66) in the healthy participants, 0.81 ng/mL (Q1: 0.37–Q3: 4.41) in those with periodontitis, and 0.54 ng/mL (Q1: 0.31–Q3: 1.45) in the group with periodontitis combined with DM. Statistical analysis revealed a significant intergroup difference in salivary apelin concentrations (*p* = 0.012) (Table 2, Figure 2). Post hoc analyses revealed that the median salivary apelin concentration in the periodontitis + DM group was significantly lower than that of the healthy group (*p* = 0.009) (Table 2, Figure 2). No significant differences were found between the other groups (*p* > 0.05). There was no statistically significant difference among the study groups regarding salivary asprosin concentrations (*p* = 0.053) (Table 2, Figure 2).

Salivary asprosin (*p* = 0.251) and apelin (*p* = 0.460) concentrations in patients with periodontitis and periodontitis + DM were compared according to the stages of periodontitis, and no statistically significant differences were observed (Table 3).

### 3.3. Spearman Correlation Analysis

Correlation outcomes between clinical periodontal parameters and salivary apelin and asprosin levels are presented in Table 4. The analysis revealed strong positive associations among the clinical parameters. PI showed significant correlations with GI (r_s_ = 0.86; *p* < 0.001), PD (r_s_ = 0.70; *p* < 0.001), and CAL (r_s_ = 0.76; *p* < 0.001). Similarly, GI was positively related to PD (r_s_ = 0.79; *p* < 0.001) and CAL (r_s_ = 0.85; *p* < 0.001). In addition, PD demonstrated a high positive correlation with CAL (r_s_ = 0.88; *p* < 0.001). Furthermore, PD showed a significant positive correlation with asprosin concentration (r_s_ = 0.29; *p* = 0.006), indicating that an increase in PD was associated with a parallel increase in asprosin concentration, while a decrease in PD corresponded to a reduction in asprosin levels. Similarly, a significant positive correlation was found between CAL and asprosin concentration (r_s_ = 0.28; *p* = 0.007). A significant negative correlation was found between PD and apelin concentration (r_s_ = −0.22; *p* = 0.035). The results indicated that an increase in PD was associated with a decrease in apelin concentration, whereas a decrease in PD corresponded to an increase in apelin levels. Similarly, a significant negative correlation was observed between CAL and apelin concentration (r_s_ = −0.25; *p* = 0.017).

### 3.4. ROC Analysis

**Figure 3:**
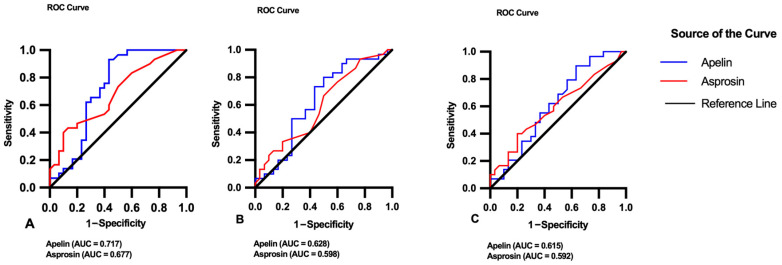
ROC curves of salivary apelin and asprosin levels for differentiating the study groups: (**A**) Healthy vs. Periodontitis + DM, (**B**) Healthy vs. Periodontitis, and (**C**) Periodontitis vs. Periodontitis + DM.

ROC curve analysis was conducted to evaluate the diagnostic performance of salivary apelin and asprosin among the study groups (Table 5, Figure 3). In the comparison between healthy and periodontitis + DM groups, apelin demonstrated acceptable discriminative ability (AUC = 0.717, 95% CI: 0.579–0.855, *p* = 0.004), while asprosin showed moderate accuracy (AUC = 0.677, 95% CI: 0.542–0.812, *p* = 0.018). In the healthy vs. periodontitis comparison, both apelin (AUC = 0.628, *p* = 0.089) and asprosin (AUC = 0.598, *p* = 0.193) exhibited limited diagnostic performance without statistical significance. Similarly, in the periodontitis vs. periodontitis + DM comparison, apelin (AUC = 0.615, *p* = 0.129) and asprosin (AUC = 0.592, *p* = 0.223) demonstrated low-to-moderate discriminative capacity, which did not reach statistical significance. Overall, apelin consistently showed slightly higher AUC values than asprosin across comparisons; however, its diagnostic performance remained within the moderate range.

## 4. Discussion

Although evidence exists regarding the physiological and pathophysiological roles of apelin and asprosin—recently identified adipokines—in diabetes and periodontitis, their functions have not yet been fully elucidated. Accordingly, the present study aimed to investigate salivary asprosin and apelin levels in periodontally healthy individuals, patients with periodontitis, and those with periodontitis and diabetes and to evaluate their relationships with clinical periodontal parameters.

Dental plaque accumulation is a major risk factor for periodontitis, and poor oral hygiene is known to substantially increase the risk of periodontal disease [42,43]. A meta-analysis reported that the PI values of individuals with periodontitis were approximately three times higher than those of periodontally healthy individuals [44]. When periodontal health deteriorates, gingival bleeding occurs due to increased inflammation in the gingival tissues. Therefore, GI values are higher in patients with gingivitis and periodontitis compared to periodontally healthy individuals [45,46]. Consistent with these findings, in our study, PI and GI values were significantly lower in the healthy group compared to the other groups. Due to the destructive nature of periodontitis, which affects the supporting structures of the teeth, PD values are significantly lower in periodontally healthy subjects than in patients with periodontitis [47,48]. A review of the literature also reveals that diabetes exacerbates periodontitis and results in higher CAL and PD values compared to individuals with periodontitis alone [49,50]. In our study, although PD and CAL values were higher in the periodontitis + DM group than in the periodontitis group, only the difference in CAL reached statistical significance (*p* = 0.019), suggesting a more pronounced impact of diabetes on clinical attachment loss.

Diabetes is widely recognized as a major contributor to the risk of developing periodontal disease. Elevated blood glucose levels may enhance gingival inflammation and contribute to the development of periodontitis [51]. Moreover, higher levels of pro-inflammatory factors in the gingival tissues of patients with uncontrolled diabetes indicate that poor glycemic control is a biological risk factor that can aggravate periodontitis [52]. Furthermore, the systemic inflammatory burden associated with periodontitis may impair diabetes control [52,53]. The connection observed between periodontal disease and DM has been further confirmed by three systematic reviews published after 2013, all of which concluded that periodontal disease exerts adverse effects on diabetes and glycemic control [54,55,56].

BMI is determined by expressing an individual’s body weight relative to the square of their height (kg/m^2^), and is commonly used to assess health risks associated with obesity [57,58]. Since the adipokines apelin and asprosin, which are secreted from adipose tissue, are also associated with obesity, obese individuals were excluded from the study design. In addition, participants were selected to have comparable BMI values, and no significant differences were observed between the groups. Several studies in the literature have detected apelin and asprosin in body fluids of non-obese individuals. Tutuş et al. identified asprosin in serum, saliva, and gingival crevicular fluid (GCF) specimens collected from individuals with periodontal health, gingivitis, and periodontitis who were not obese [59]. Similarly, Yoldaş et al. detected apelin in the GCF of three groups: periodontally healthy, gingivitis, and periodontitis subjects [60].

Apelin, a peptide adipokine, has recently been identified as a promising therapeutic target due to its regulatory effects in metabolic disorders such as diabetes and obesity [61]. Experimental data indicate that the administration of apelin confers protective benefits in animal models of obesity and diabetes [62]. In addition, clinical research has reported notable variations in circulating apelin concentrations between diabetic and/or obese individuals and healthy controls, suggesting that apelin may participate in the pathophysiology of these conditions and serve as a potential biomarker for metabolic dysregulation [63].

According to our results, salivary apelin levels were lower in individuals with periodontitis + DM compared to those with periodontitis alone; however, this difference was not statistically significant. Similarly, periodontitis patients exhibited lower salivary apelin levels than periodontally healthy individuals, although the difference was also not significant. The only statistically significant difference among the groups was observed between the periodontitis + DM group and the periodontally healthy group. Nevertheless, our correlation analysis revealed a significant association between clinical periodontal parameters and apelin levels. These findings suggest that the variations observed in our results may be attributed to the involvement of apelin in the shared pathogenesis of diabetes and periodontitis. However, further studies with larger sample sizes are required to confirm this association.

Findings regarding apelin levels in diabetic individuals are inconsistent in the literature. Erdem et al. reported that serum apelin concentrations were markedly lower in individuals recently diagnosed with diabetes than in those without the disease [64]. In a Chinese cohort, researchers observed that plasma apelin concentrations were reduced in newly diagnosed and untreated individuals with type 2 diabetes when contrasted with non-diabetic participants [65]. Likewise, Onalan et al. found markedly lower serum apelin levels among patients exhibiting impaired glucose tolerance, metabolic syndrome, or type 2 diabetes when compared with the control group [66]. Within the present research, the apelin concentration reached its lowest value in the periodontitis + DM group, exhibiting a statistically meaningful variation relative to the periodontally healthy participants. These findings align closely with previous observations. Some studies have also reported higher plasma apelin levels in diabetic individuals [67,68,69]. Sun et al., in a meta-analysis comparing women with gestational diabetes to those without the condition, reported no statistically significant variation in apelin concentrations [70]. A review of the literature indicates that the variability in findings may depend on factors such as whether diabetes is newly diagnosed or untreated, the presence of diabetic complications, the type of diabetes, and the stage of the disease. This variability suggests that the role of apelin as a biomarker in diabetes is complex and context-dependent. To eliminate potential confounding effects of diabetes type and complications, our study included only patients with type 2 diabetes and HbA1c levels between 7 and 8.5.

Beyond its potential role as a biomarker, experimental studies have also explored the functional role of apelin in periodontal tissues. Ziskoven et al. (2024) [71] showed that apelin alleviated the suppressive impact of *Fusobacterium nucleatum*—a recognized periodontal pathogen—on PDL cell migration. Based on these findings, the authors proposed that apelin could function as a regulatory factor in the development of periodontitis [71]. Conversely, in another study by the same research group in 2023, apelin was found to further enhance the production of pro-inflammatory and proteolytic molecules induced by *F. nucleatum* [72]. As seen, studies investigating whether apelin exerts beneficial or detrimental effects on periodontal tissues are limited, and their results remain inconsistent. However, these findings suggest that there may be a potential link between apelin and the pathogenesis of periodontitis.

Koguchi et al. described apelin as an anti-inflammatory cytokine and demonstrated that it suppresses key inflammatory mediators such as TNF-α and IL-1β [73]. Prasanna et al. reported a negative correlation between serum apelin levels and PD and CAL indices and showed that both serum and salivary apelin levels were lower in patients with periodontitis compared to healthy controls. In addition, apelin levels have been reported to increase in obese individuals, with a more pronounced elevation observed in cases where obesity is accompanied by T2DM [74]. These findings are consistent with our results, which demonstrate decreased apelin levels in periodontitis and an inverse relationship with clinical periodontal parameters. Taken together, these findings suggest that apelin may function as an anti-inflammatory molecule in periodontal tissues. This interpretation is supported by studies reporting higher levels of anti-inflammatory cytokines such as IL-10, IL-4, and IL-11 in periodontally healthy individuals [75,76,77]. However, the current evidence is not sufficient to definitively establish this role, and this interpretation should be considered speculative. Well-designed future studies are needed to clarify the exact role of apelin in periodontal disease.

The inconsistency of findings regarding apelin levels in the literature may be attributed to its dual nature, exhibiting both anti-inflammatory and pro-inflammatory properties. The reduced apelin levels observed in periodontitis may be explained by an inadequate apelin response during local inflammation or by its consumption in the presence of increased inflammatory activity. The varying results reported in diabetic patients may be influenced by factors such as obesity status, the presence of diabetic complications, and coexisting metabolic syndrome.

In this context, heterogeneity in study populations and the influence of systemic factors may explain the conflicting findings reported in the literature. Our study is particularly important in that it was conducted in a population in which confounding factors such as obesity and age were excluded, and it demonstrates decreased apelin levels in the presence of periodontitis and diabetes.

Asprosin has attracted interest in diabetes due to its role in glucose regulation and its association with insulin resistance and metabolic disturbances [32]. Accordingly, numerous studies have evaluated plasma asprosin levels in diabetic individuals in relation to both disease complications and treatment. Certain reports have indicated that circulating asprosin levels are elevated in patients with diabetes compared to those observed in healthy controls [78,79]. Others found no significant difference between diabetic and healthy subjects; however, serum asprosin levels were significantly higher in patients with diabetic complications than in both healthy and diabetic groups [80,81]. Multiple studies conducted on diabetic rats and humans have demonstrated that diabetes treatment significantly reduces serum asprosin levels and decreases the expression of the FBN1 gene, which encodes asprosin [82,83,84,85]. This reduction may be associated with improved glycemic control and insulin sensitivity. Zhang et al. reported that, after adjusting for age and sex, asprosin was not an ideal predictor for type 2 diabetes [86]. According to the results of our study, no significant difference was observed in salivary asprosin levels between the diabetic group and other groups. This finding may be related to the fact that individuals with diabetic complications were not included in the study, and HbA1c levels were limited to the range of 7–8.5. Additionally, the fact that our diabetic participants were under medical treatment and that obesity was excluded from the study population may have also influenced these results.

In the study conducted by Tutuş et al., salivary asprosin levels were found to be statistically higher in the gingivitis and periodontitis groups compared to healthy individuals [59]. In our study, salivary asprosin levels were similar among the three groups, and therefore, our findings are not consistent with that research.

According to our correlation results, salivary asprosin showed a direct relationship with PD and CAL values. Similarly, in a study conducted by Gül et al., a significant positive relationship was also found between serum and salivary asprosin levels and the PD and CAL indices [87]. In another study, serum asprosin levels decreased in periodontitis-induced rats treated with eugenol; however, it could not be determined whether this decrease was caused by the direct effect of eugenol on asprosin or by the improvement of periodontitis through treatment [88].

In this study, no significant differences were observed in salivary asprosin levels among the groups; however, a positive correlation was found between salivary asprosin levels and PD and CAL. This finding suggests that asprosin may be associated with the severity of periodontal tissue destruction rather than categorical group differences. The moderate discriminative performance observed in ROC analysis only between healthy individuals and the periodontitis+DM group may be explained by the greater contrast between the extreme groups rather than a diabetes-specific effect; notably, no significant difference was detected between the periodontitis and periodontitis+DM groups.

The absence of significant intergroup differences may be attributed to several factors, including the limited sample size, cross-sectional design, exclusion of diabetic complications, restriction of HbA1c values within a specific range, and the exclusion of obese individuals. These factors may have resulted in a metabolically more homogeneous study population, thereby limiting the ability to detect potential differences between groups.

Nevertheless, the biological basis of the observed association between asprosin levels and periodontal clinical parameters remains unclear. It is not evident whether this relationship reflects systemic inflammatory processes or local production within periodontal tissues. Further studies using serum and GCF samples, with larger sample sizes and broader metabolic profiles, are needed to clarify this relationship.

Among the strengths of our study are the well-designed study groups, the meticulous recording of clinical indices, and the careful execution of saliva collection and biochemical analysis procedures. Additionally, the absence of differences between the groups in terms of age, gender, and BMI is also one of the strong aspects of our study.

There are some limitations to our study. Although confounding factors such as smoking, obesity, age, and gender were excluded, non-modifiable risk factors such as genetics remain among the limitations. In addition, the sample size and cross-sectional study design limit the possibility of longitudinal evaluation. The assessment of only salivary levels, without considering serum and GCF levels, restricts the interpretation of their relationship with periodontal disease. Moreover, since our study is observational in nature, a causal relationship between salivary apelin and asprosin levels and the pathophysiological mechanisms of type 2 diabetes and periodontitis could not be confirmed.

According to our correlation results, no association was observed between asprosin and apelin. However, asprosin showed a positive correlation with PD and CAL, whereas apelin demonstrated a negative correlation with these parameters, suggesting potentially distinct roles of these adipokines in periodontal inflammation. The significantly lower salivary apelin levels observed in periodontitis+DM patients suggest that apelin may have potential as a non-invasive biomarker candidate for distinguishing diabetic periodontitis from periodontal health. Considering previously reported molecular interactions of apelin and asprosin in metabolic and inflammatory pathways, these findings further underscore their possible relevance in the interplay between periodontal and metabolic diseases. Nevertheless, further longitudinal studies are required to confirm its clinical applicability and biological significance.

## 5. Conclusions

In the present study, salivary apelin levels were lowest in the DM + periodontitis group and significantly lower compared to the healthy group. PD and CAL indices showed a positive correlation with salivary asprosin and a negative correlation with salivary apelin. The differing correlations of apelin and asprosin with periodontal parameters may suggest distinct roles of these adipokines in periodontal inflammation; however, further studies are required to fully elucidate the nature and clinical significance of these relationships. Furthermore, apelin may serve as a potential biomarker in diabetes. Nevertheless, additional research is required to clarify the mechanisms by which apelin and asprosin adipokines contribute to the interaction among diabetes, periodontitis, and obesity.

## Figures and Tables

**Table 1:** Demographic characteristics, clinical periodontal parameters, and salivary apelin and asprosin concentrations of the study participants.

Parameter	All Participants
Gender (male/female) *n* (%)	42 (46.7%)/48 (53.3%)
Age (years) (mean ± SD)	41.9 ± 3.2
Periodontal status (*n* (%)) (Healthy/periodontitis/periodontitis + dm)	30 (33.3)/30 (33.3)/30 (33.3)
Plaque index (PI) (mean ± SD)	1.39 ± 0.67
Gingival index (GI) (mean ± SD)	1.11 ± 0.87
Probing depth (PD) (mean ± SD)	2.26 ± 0.94
Clinical attachment loss (CAL) (mean ± SD)	2.64 ± 2.04
Apelin concentration (ng/mL) (median (Q1–Q3))	0.727 (0.338–4.592)
Asprosin concentration (ng/mL) (median (Q1–Q3))	0.083 (0.05–0.14)
BMI (kg/m^2^) (mean ± SD)	24.14 ± 1.28

Data are expressed as mean ± standard deviation or median (Q1–Q3) and *n* (%), depending on the distribution of the variables.

**Table 2:** Comparison of demographic characteristics, clinical periodontal parameters, and salivary apelin and asprosin concentrations among the study groups.

	Study Groups				Subgroup Analysis		
Parameter	Healthy	Periodontitis	Periodontitis + DM	*p*	Healthy-Periodontitis *p*	Healthy-Periodontitis + DM *p*	Periodontitis-Periodontitis + DM *p*
Gender (male/female) *n* (%)	14 (46.7)/16 (53.3)	14 (46.7)/16 (53.3)	14 (46.7)/16 (53.3)	1.000 ^a^	**-**	**-**	**-**
Age (years) (median (Q1–Q3))	41.43 (36–46)	41.43 (36–49)	42.8 (38–47)	0.158 ^b^	0.864	0.084	0.116
Apelin concentration (ng/mL) (median (Q1–Q3))	4.56 (0.36–38.66)	0.81 (0.37–4.41)	0.54 (0.31–1.45)	**0.012 ^b^**	**-**	**0.009**	**-**
Asprosin concentration (ng/mL) (median (Q1–Q3))	0.07 (0.04–0.1)	0.08 (0.06–0.14)	0.1 (0.06–0.26)	0.053 ^b^	-	-	-
BMI (kg/m^2^) (median (Q1–Q3))	23.85 (23.5–24.68)	24.40 (23.7–24.68)	24.35 (23.53–24.80)	0.645 ^b^	-	-	**-**
Plaque index (PI) (median (Q1–Q3))	0.66 (0.46–0.78)	1.74 (1.47–2.11)	1.73 (1.43–2.18)	**<0.001 ^b^**	**<0.001**	**<0.001**	**-**
Gingival index (GI) (median (Q1–Q3))	0 (0–0.4)	1.44 (1.25–1.85)	1.59 (1.17–2.01)	**<0.001 ^b^**	**<0.001**	**<0.001**	**-**
Clinical attachment loss (CAL) (mm) (median(Q1–Q3))	-	3.38 (2.98–4.25)	4.22 (3.61–4.62)	**0.019** **^c^**	**-**	**-**	**-**
Probing depth (PD) (mm) (median (Q1–Q3))	1.44 (1.36–1.54)	2.19 (1.84–2.84)	2.7 (2.16–3.24)	**<0.001 ^b^**	**<0.001**	**<0.001**	**-**

Data are expressed as median (Q1:Q3) and *n*%. ^a^: Pearson’s chi-square test, ^b^: Kruskal–Wallis test, ^c^: Mann–Whitney U test. Statistically significant p-values (*p* < 0.05) are shown in bold.

**Table 3:** Comparison of salivary apelin levels according to periodontitis stages: asprosin (*n*:60); apelin (*n*:59).

	Salivary Asprosin Levels	Salivary Apelin Levels
Stage II (median (Q1–Q3))	0.08 (0.05–0.11)	0.49 (0.31–2.37)
Stage III (median (Q1–Q3))	0.1 (0.07–0.26)	0.58 (0.24–2.01)
Stage IV (median (Q1–Q3))	0.14 (0.06–0.19)	1.26 (0.51–2.22)
*p*	0.251 ^a^	0.460 ^a^

Data are expressed as median (Q1:Q3) and n%. ^a^: Kruskal–Wallis test.

**Table 4:** Correlation of clinical periodontal parameters, saliva apelin and asprosin concentrations..

	PI		GI		PD		CAL		Asprosin		Apelin	
	r_s_	*p*	r_s_	*p*	r_s_	*p*	r_s_	*p*	r_s_	*p*	r_s_	*p*
PI	-	-	-	-	-	-	-	-	-	-	-	-
GI	0.86	**<0.001**	-	-	-	-	-	-	-	-	-	-
PD	0.70	**<0.001**	0.79	**<0.001**	-	-	-	-	-	-	-	-
CAL	0.76	**<0.001**	0.85	**<0.001**	0.88	**<0.001**	-	-	-	-	-	-
Asprosin	0.18	0.089	0.17	0.118	0.29	**0.006**	0.28	**0.007**	-	-	-	-
Apelin	−0.17	0.118	−0.20	0.058	−0.22	**0.035**	−0.25	**0.017**	0.03	0.818	-	-

r_s_: Spearman correlation coefficient. Bold values indicate statistical significance (*p* < 0.05)

**Table 5:** Diagnostic accuracy of salivary apelin and asprosin in differentiating study groups: ROC curve analysis.

Groups	Salivary Biomarkers	Cut-Off (ng/mL)	AUC (95% CI)	Sensitivity (%)	Specificity (%)	*p* Value
Healthy vs. Periodontitis + DM	Apelin	0.67	0.717 (0.579–0.855)	65.5	70.0	0.004 **
	Asprosin	0.075	0.677 (0.542–0.812)	66.7	53.3	0.018 *
Healthy vs. Periodontitis	Apelin	2.847	0.628 (0.483–0.773)	73.3	56.7	0.089
	Asprosin	0.065	0.598 (0.454–0.742)	66.7	50.0	0.193
Periodontitis vs. Periodontitis + DM	Apelin	0.595	0.615 (0.470–0.760)	62.1	56.7	0.129
	Asprosin	0.155	0.592 (0.447–0.736)	40.0	80.0	0.223

AUC: area under the curve; CI: confidence interval; DM: diabetes mellitus; * *p* < 0.05; ** *p* < 0.01.

## Data Availability

The datasets generated and/or analyzed during the current study are provided in the supplementary statistical table, which includes demographic characteristics (age, gender), clinical periodontal parameters, BMI values, and salivary apelin and asprosin concentrations (ng/mL). Raw measurement data are not publicly available due to confidentiality restrictions, but all processed datasets used for statistical analyses are fully included in this submission and are available from the corresponding author upon reasonable request.

## Supplementary Materials

The following supporting information can be downloaded at: https://www.mdpi.com/article/10.3390/diagnostics16071054/s1, Table S1: Raw data of salivary apelin and asprosin levels in all study groups.

## Abbreviations

DM	Diabetes Mellitus
BMI	Body Mass Index
PI	Plaque Index
GI	Gingival Index
PD	Probing Depth
CAL	Clinical Attachment Loss
GCF	Gingival Crevicular Fluid
ELISA	Enzyme-Linked Immunosorbent Assay
